# Genes regulated by SATB2 during neurodevelopment contribute to schizophrenia and educational attainment

**DOI:** 10.1371/journal.pgen.1007515

**Published:** 2018-07-24

**Authors:** Laura Whitton, Galina Apostolova, Dietmar Rieder, Georg Dechant, Stephen Rea, Gary Donohoe, Derek W. Morris

**Affiliations:** 1 Cognitive Genetics and Cognitive Therapy Group, Neuroimaging, Cognition and Genomics (NICOG) Centre and NCBES Galway Neuroscience Centre, School of Psychology and Discipline of Biochemistry, National University of Ireland Galway, Galway, Ireland; 2 Institute for Neuroscience, Medical University of Innsbruck, Innsbruck, Austria; 3 Centre for Chromosome Biology, Discipline of Biochemistry, National University of Ireland Galway, Galway, Ireland; Albert Einstein College of Medicine, UNITED STATES

## Abstract

*SATB2* is associated with schizophrenia and is an important transcription factor regulating neocortical organization and circuitry. Rare mutations in *SATB2* cause a syndrome that includes developmental delay, and mouse studies identify an important role for *SATB2* in learning and memory. Interacting partners *BCL11B* and *GATAD2A* are also schizophrenia risk genes indicating that other genes interacting with or are regulated by SATB2 are making a contribution to schizophrenia and cognition. We used data from *Satb2* mouse models to generate three gene-sets that contain genes either functionally related to SATB2 or targeted by SATB2 at different stages of development. Each was tested for enrichment using the largest available genome-wide association studies (GWAS) datasets for schizophrenia and educational attainment (EA) and enrichment analysis was also performed for schizophrenia and other neurodevelopmental disorders using data from rare variant sequencing studies. These SATB2 gene-sets were enriched for genes containing common variants associated with schizophrenia and EA, and were enriched for genes containing rare variants reported in studies of schizophrenia, autism and intellectual disability. In the developing cortex, genes targeted by SATB2 based on ChIP-seq data, and functionally affected when SATB2 is not expressed based on differential expression analysis using RNA-seq data, show strong enrichment for genes associated with EA. For genes expressed in the hippocampus or at the synapse, those targeted by SATB2 are more strongly enriched for genes associated EA than gene-sets not targeted by SATB2. This study demonstrates that single gene findings from GWAS can provide important insights to pathobiological processes. In this case we find evidence that genes influenced by SATB2 and involved in synaptic transmission, axon guidance and formation of the corpus callosum are contributing to schizophrenia and cognition.

## Author summary

Schizophrenia is a complex disorder caused by many genes. Using new gene discoveries to understand pathobiology is a foundation for development of new treatments. Current drugs for schizophrenia are only partially effective and do not treat cognitive deficits, which are key factors for explaining disability, leading to unemployment, homelessness and social isolation. Genome-wide association studies (GWAS) of schizophrenia have been effective at identifying individual SNPs and genes that contribute to risk but have struggled to immediately uncover the bigger picture of the underlying biology of the disorder. Here we take an individual gene identified in a schizophrenia GWAS called *SATB2*, which on its own is a very important regulator of brain development. We use functional genomics data from mouse studies to identify sets of others genes that are influenced by *SATB2* during development. We show that these gene sets are enriched for common variants associated with schizophrenia and educational attainment (used as a proxy for cognition), and for rare variants that increase risk of various neurodevelopmental disorders. This study provides evidence that the molecular mechanisms that underpin schizophrenia and cognitive function include disruption of biological processes influenced by *SATB2* as the brain is being organized and wired during development.

## Introduction

Neocortical organization and circuitry requires the coordinated execution of a series of developmental processes, including the specification of neuronal identity, neuronal migration, and wiring of neural circuits [1]. Special AT-rich sequence-binding protein 2 (*SATB2*) and B-cell lymphoma/leukaemia 11B (*BCL11B*) are two of the several key transcription factors that control the projection identity of cortical neurons (subcortical vs. callosal) during cortical development[2]. SATB2 modifies higher-order chromatin structure and can mediate chromatin loop formation via self-association in order to regulate other genes [3–6]. *De novo* structural and point mutations in *SATB2* result in *SATB2* haploinsufficiency and SATB2-associated syndrome, which is characterised by developmental delay, mild to severe intellectual disability, speech and behavioural problems and abnormal craniofacial features [7].

During development, pyramidal neurons (excitatory projection neurons primarily found in the cerebral cortex [8]) project axons across multiple brain regions and to the corticospinal tract [9]. Based on their projections, pyramidal neurons can be divided into two groups; deep layer neurons (located in cortical layers 5 and 6) projecting to subcortical regions and upper layer neurons (located in cortical layers 2, 3 and 4) projecting across the corpus callosum to the contralateral hemisphere[10]. SATB2 is required for the projection of upper layer neurons and loss of SATB2 leads to upper layer neurons incorrectly projecting to subcortical regions [11,12]. In the adult CNS, SATB2 is critically important as a regulator of synaptic plasticity in the hippocampus that underlies memory functions [13,14].

SATB2 specifically mediates callosal projection identity by repressing the expression of BCL11B (also known as CTIP2), a zinc finger protein required for subcortical projection neuron identity [2,11]. SATB2 directly binds to the *BCL11B* locus and recruits the Ski protein and the nucleosome remodeling deacetylase (NuRD) complex to initiate chromatin modifications inhibiting *BCL11B* expression [12,15]. BCL11B is required for the postnatal development of the hippocampus and its loss leads to impaired hippocampal learning and memory in the adult brain [13,16]. GATA zinc finger domain containing 2A (*GATAD2A*; also known as P66-alpha), is a core component of the NuRD complex and mediates the interaction between histones and other core proteins [17,18]. GATAD2A plays a key role in memory preservation through activity-induced histone modifications[19].

Analysis of just genome-wide significant SNPs for SZ implicated *SATB2*, *BCL11B* and *GATAD2A* in the aetiology of this disorder as epigenetic regulators of neocortical development [20]. We hypothesized that variation in other genes that function with or are regulated by *SATB2* are also contributing to SZ aetiology. Given the high polygenicity of SZ and the weak individual SNP effects detected in GWAS, we decided to move beyond individual SNP analysis and instead performed gene-set analysis (GSA) on three gene-sets that contain genes either functionally related to SATB2 or targeted by SATB2 at different stages of development. This makes it possible to detect the effects of multiple weaker associations that may be missed by individual SNP or gene based-analysis.

We tested these SATB2 gene-sets for a contribution to SZ using the largest available GWAS dataset that used 40,675 cases and 64,643 controls [21]. Given the genetic overlap between SZ and cognition [22], and the facts that SATB2 has an identified role in memory function[13,14] and that cognitive deficits are present in individuals with SATB2 syndrome[7], we investigated these gene-sets for a genetic contribution to cognition. We based this analysis on educational attainment (EA), a proxy for cognition based on measuring years of schooling, using the largest available EA GWAS results from 328,917 samples[23]. We also sought independent evidence that these gene-sets contribute to SZ and other neurodevelopmental disorders with cognitive deficits by testing these gene-sets for enrichment of genes that contain *de novo* variants and genes with an increased burden of ultra-rare protein altering variants in SZ cases.

## Results

### Generation of SATB2 gene-sets

We developed three different gene-sets containing genes that either function together with or are regulated by murine Satb2 at different stages of development. The first gene-set contains 127 genes (S1 Table), the majority of which (n = 117) are genes that have been reported as differentially expressed in the cortices of Satb2 mutant mice during neurodevelopment [11,24]. Additionally, the gene-set contains genes considered to be vital components of the NuRD complex [25] as it has been previously shown to facilitate Satb2-mediated repression of Bcl11b during development. This first gene-set is called SATB2+NuRD. The second gene-set is based on data from a single study that generated a dataset of 1,341 ChIP-seq peaks that map binding sites of SATB2 in cortices of wild type mice at embryonic day (E) 15.5 [24]. By mapping these ChIP-seq peaks to regulatory regions of genes, we generated a set of 778 genes that are targets of and potentially regulated by SATB2 during cortical development. This second gene-set is called SATB2_Cort (S2 Table). The third gene-set is based on data from a single study that generated a dataset of 5,027 ChIP-seq peaks that map binding sites of Satb2 in primary hippocampal cell cultures from wild type mice at postnatal day P0 to P1 [13]. We mapped these ChIP-seq peaks to identify 4,138 target genes and called this gene-set SATB2_Hipp (S3 Table). Full details on the generation of each gene-set are supplied in Materials and Methods.

The rationale for three rather than a single gene-set is as follows: SATB2+NuRD includes genes that were reported in a number of different studies that used different mouse models and study designs. SATB2_Cort and SATB2_Hipp are based on single studies each using material from different brain regions at different time points during development and we know that SATB2 has different functions at different stages of development. Combining the three into a single gene-set would miss the opportunity to test for enrichment in SZ and EA GWAS data in these spatially and temporally defined gene-sets that capture SATB2 function at important brain regions and different developmental time points. S1 Fig shows the overlap between the three gene-sets. Seven genes are common to all three gene-sets. Gene symbols from each overlapping category are listed in S4 Table.

### Analysis of SATB2+NuRD gene-set

We used MAGMA [26] for GSA to simultaneously study multiple genetic markers in order to determine their joint effect and test if the genes in SATB2+NuRD were more strongly associated with SZ or EA than other genes in the genome. MAGMA uses summary statistics (SNP P values) from GWAS and a significant enrichment within SATB2+NuRD points to variation across those genes influencing SZ and/or EA, and provides further evidence that biological functions related to SATB2 are part of disorder aetiology. The SATB2+NuRD gene-set (n = 127 genes) was enriched for SZ risk genes (P = 9.54x10^-5^) and for genes associated with EA (P = 0.0005). We knew SATB2+NuRD contained three genes associated with SZ (*SATB2*, *BCL11B* and *GATAD2A*). To test if these three genes were driving the significant enrichment in SZ, we removed them and re-ran the GSA in the SZ data using a smaller SATB2+NuRD gene-set (n = 124). We still detected enrichment of SZ (P = 0.001) indicating this gene-set contains multiple other genes associated with SZ.

Brain-expressed genes are a major contributor to SZ [27] and EA [23]. It is possible that the enrichment detected here could be due to the SATB2+NuRD gene-set representing a set of brain-expressed genes. However, the SATB2+NuRD enrichment was robust to the inclusion in the analyses of both ‘brain-expressed’ (n = 14,243) and ‘brain-elevated’ (n = 1,424) gene-sets as covariates (SZ: P = 0.0003 and P = 0.0005 respectively; EA: P = 0.0004 and P = 0.0007 respectively). To examine if the enrichment we detect for SZ and EA is a property of polygenic phenotypes in general, we obtained GWAS summary statistics for 10 phenotypes and we tested SATB2+NuRD for enrichment in each one. These were child-onset psychiatric disorders (attention deficient hyperactivity disorder (ADHD) and autism spectrum disorder (ASD)), adult-onset psychiatric disorders (bipolar disorder (BPD) and obsessive compulsive disorder (OCD)), other brain-related disorders (Alzheimer’s disease (AD) and stroke (STR)), and non-brain related diseases (cardiovascular disease (CAD), Crohn’s disease (CD), ulcerative colitis (UC) and type 2 diabetes (T2D)). SATB2+NuRD was not enriched for any of the 10 phenotypes (S2 Fig).

### Analysis of SATB2_Cort gene-set

The SATB2_Cort gene-set (n = 778 genes) was enriched for EA genes (P = 0.0068) but not for SZ risk genes (P = 0.26). The enrichment in SATB2_Cort for EA was robust to the inclusion of both ‘brain-expressed’ and ‘brain-elevated’ gene-sets as covariates (P = 0.013 and P = 0.0077 respectively). When tested for enrichment in 10 other GWAS datasets, SATB2_Cort only showed one nominally significant enrichment (for ADHD; P = 0.021) but this did not survive multiple test correction (S3 Fig).

**Fig 1:**
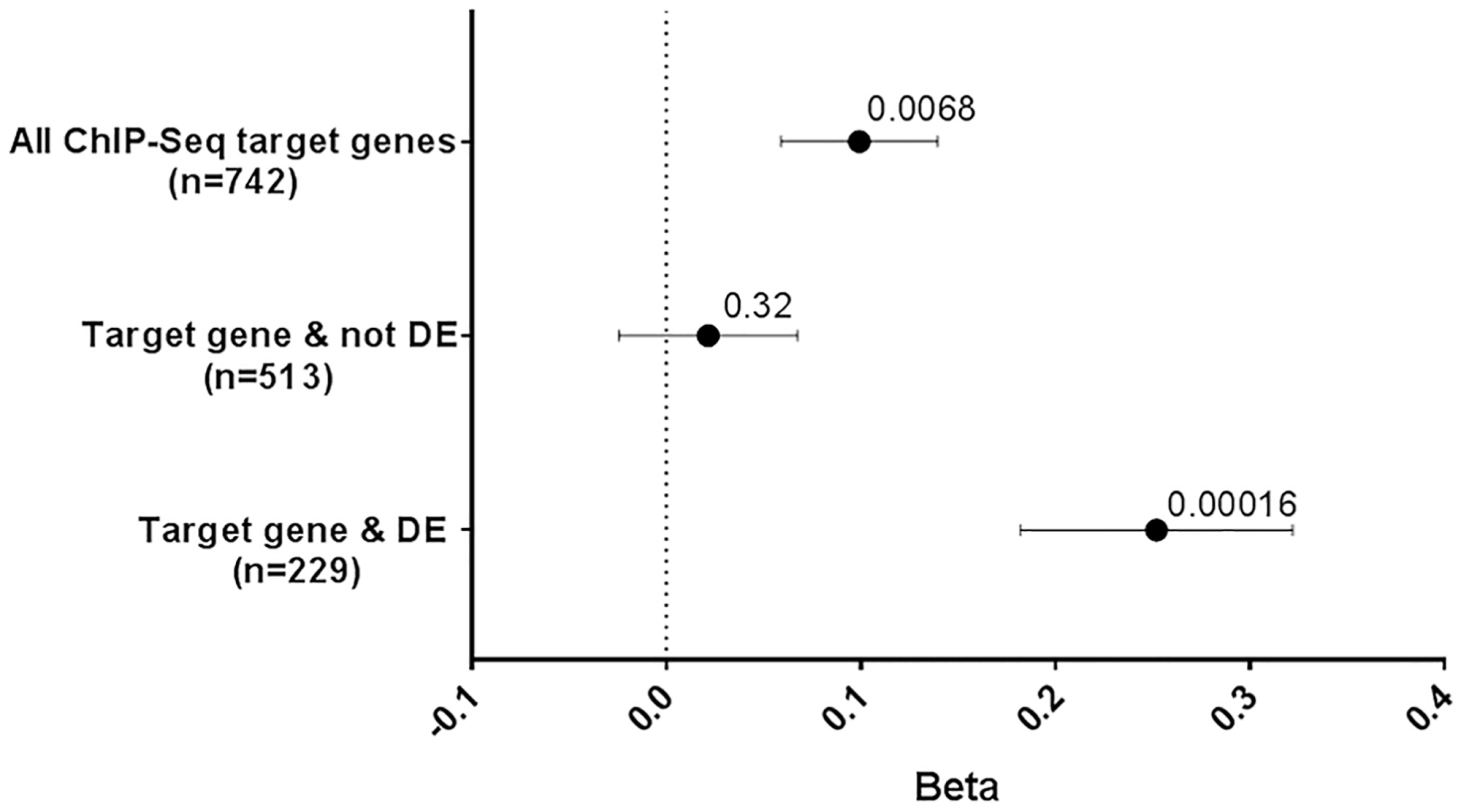
GSA of SATB2_Cort in EA, and the partition of these SATB2 target genes into those that were or were not differentially expressed (DE) in P0 cortices of SATB2 WT v KO mice. Gene-sets and number of genes are plotted on the y-axis. Beta values (effect sizes) as calculated by MAGMA are plotted on the x-axis with P-values shown above each data point. Horizontal bars indicate standard error. The overall enrichment signal (top) appears to be driven by those genes that were DE (bottom), indicating that of genes targeted and potentially regulated by SATB2, it is those functionally impacted by its loss that contribute more to EA.

The study that reported the SATB2 ChIP-Seq data, which we used to map the SATB2 target genes in the SATB2_Cort gene-set, also reported 3,129 genes that were differentially expressed in P0 cortices of SATB2 wild-type (WT) v knock-out (KO) mice. We used these data to identify those SATB2 target genes that were differentially expressed and thus functionally impacted by the loss of SATB2. Fig 1 shows that the subset of genes within SATB2_Cort that are differentially expressed are making a stronger contribution to EA (n = 229 genes; P = 0.00016) than those genes that are not differentially expressed (n = 513 genes; P = 0.32). Thus, variation in genes that are both targeted by SATB2 and functionally affected when SATB2 is not expressed in the mouse cortex contributes to EA in the general population.

Given SATB2’s role in the formation and structural integrity of the cerebral cortex [28], we tested our SATB2_Cort gene-set for enrichment of genes against intracranial volume [29]. Intracranial volume was chosen because it is closely related to brain volume in early life as the brain develops after which it becomes stable when the brain has fully developed and it remains unaffected by later age-related changes [30,31]. SATB2_Cort was not enriched for genes associated with intracranial volume and no enrichment was observed for the subset of genes within SATB2_Cort that showed the strongest enrichment for EA, i.e. those genes that were differentially expressed in the mouse cortex upon SATB2 ablation (S5 Fig).

### Analysis of SATB2_Hipp gene-set

The SATB2_Hipp gene-set (n = 4,138 genes) was enriched for SZ risk genes (P = 0.0040) and for EA genes (P = 2.03x10^-6^). The enrichment in SATB2_Hipp for SZ did not remain significant when we conditioned on the set of ‘brain-expressed’ genes (P = 0.058). The enrichment in SATB2_Hipp for EA was robust to the inclusion of both ‘brain-expressed’ and ‘brain-elevated’ gene-sets as covariates (P = 3.77x10^-5^ and P = 5.74x10^-6^ respectively). When tested for enrichment in 10 other GWAS datasets, SATB2_Hipp only showed two nominally significant enrichments (for ASD (P = 0.028) and CAD (P = 0.040) but these did not survive multiple test correction (S4 Fig).

**Fig 2:**
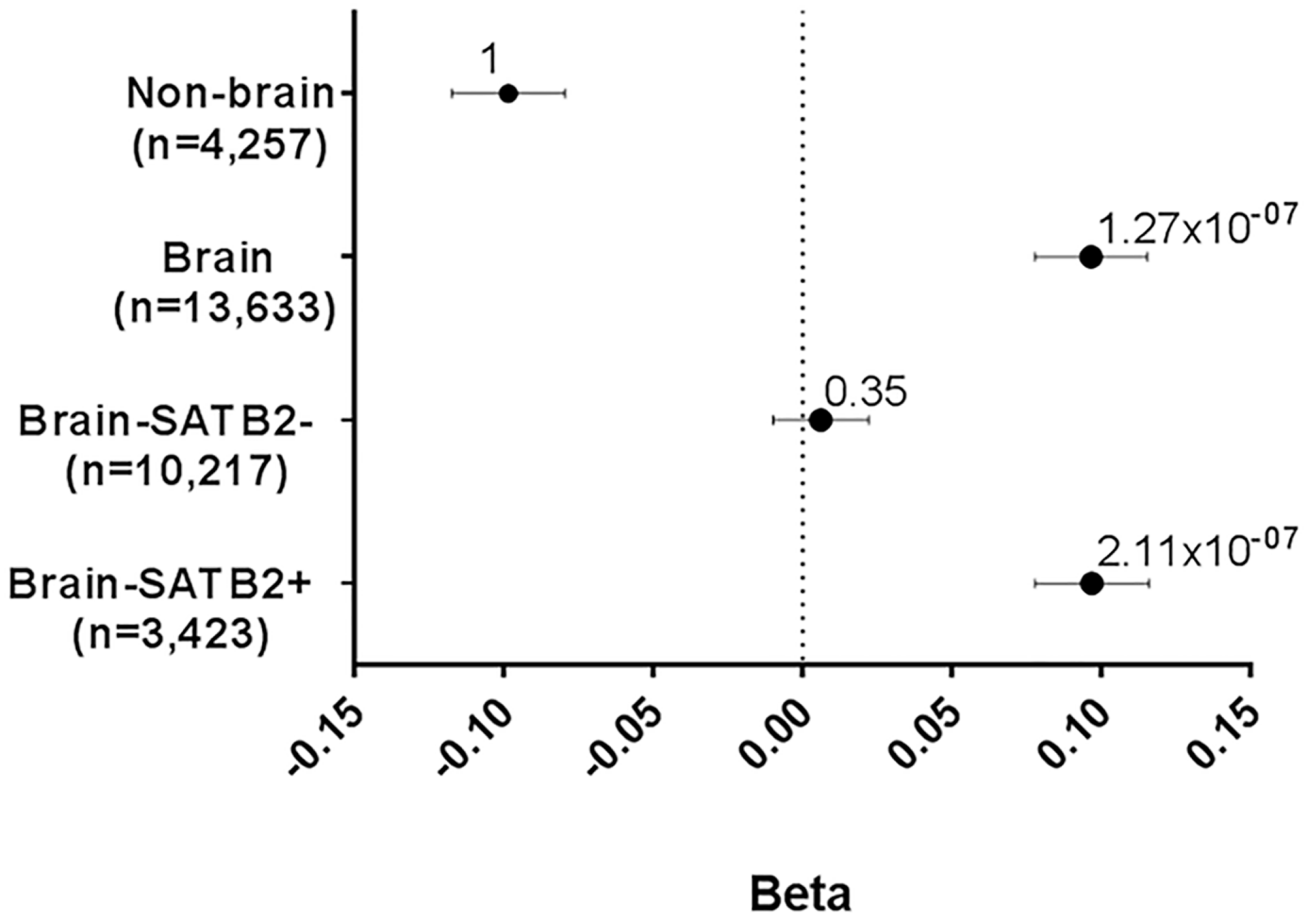
GSA of SATB2_Hipp in EA in brain. Gene-sets and number of genes are plotted on the y-axis. Beta values (effect sizes) as calculated by MAGMA are plotted on the x-axis with P-values shown above each data point. Horizontal bars indicate standard error. Analysis of non-brain and brain expressed genes, with the latter partitioned into SATB2+ or SATB2- based on genes being present in the SATB2_Hipp gene-set or not.

**Fig 3:**
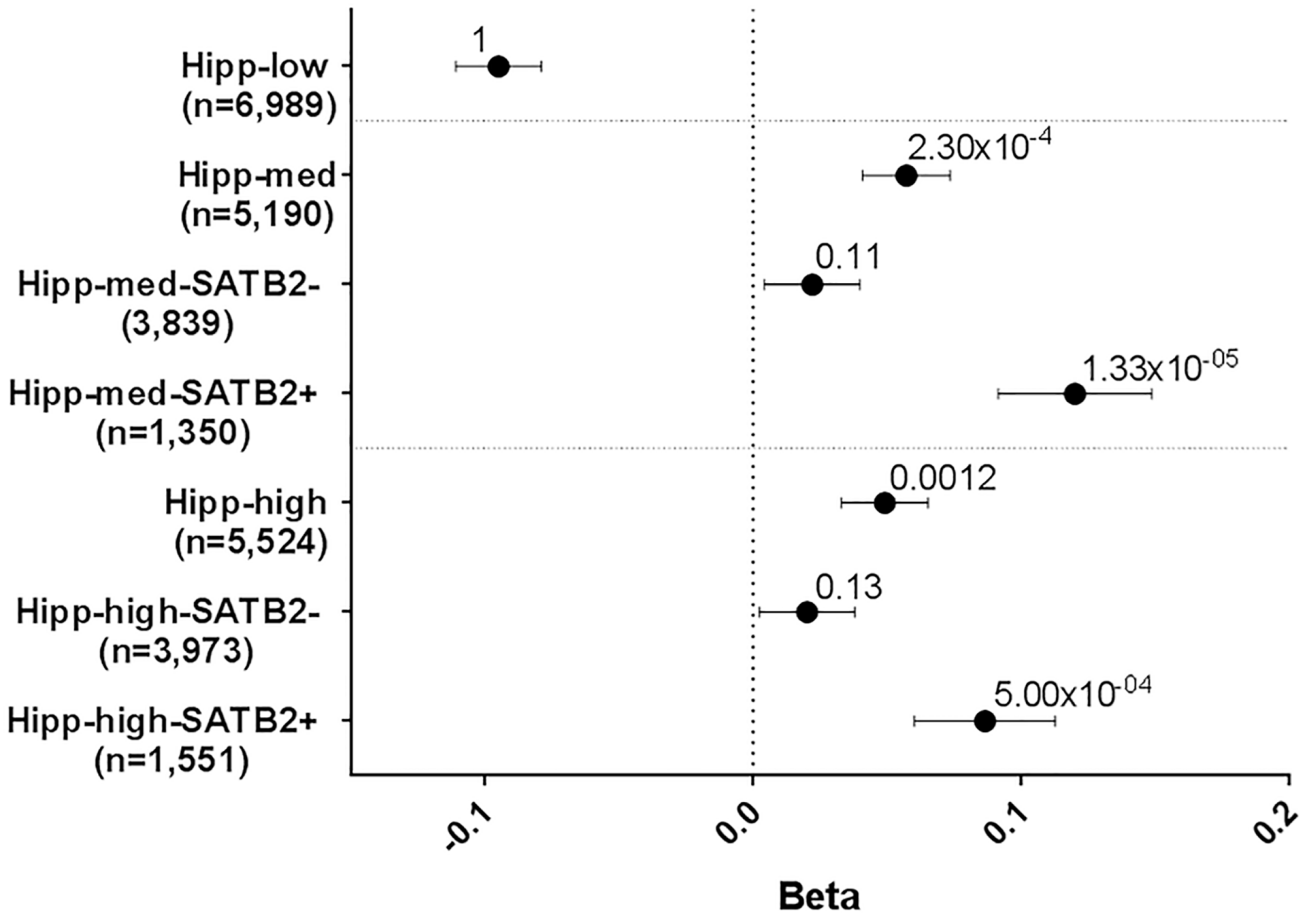
GSA of SATB2_Hipp in EA in hippocampus (Hipp). Gene-sets and number of genes are plotted on the y-axis. Beta values (effect sizes) as calculated by MAGMA are plotted on the x-axis with P-values shown above each data point. Horizontal bars indicate standard error. Genes categorized as low, medium (med) or high expressed based on their expression in the human hippocampus at pcw 37–1 year and partitioned into SATB2+ and SATB- based on genes being present in the SATB2_Hipp gene-set or not.

**Fig 4:**
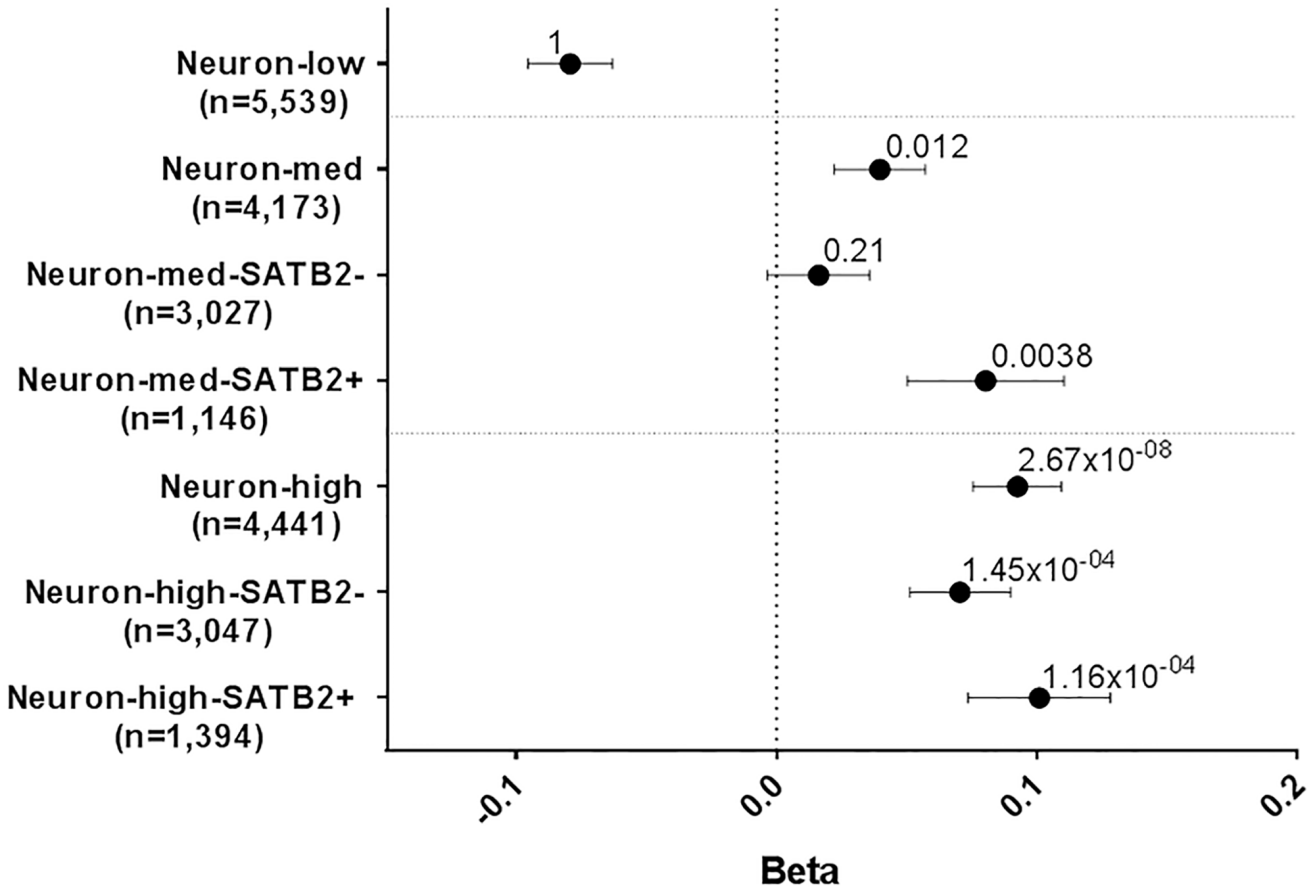
GSA of SATB2_Hipp in EA in neurons. Gene-sets and number of genes are plotted on the y-axis. Beta values (effect sizes) as calculated by MAGMA are plotted on the x-axis with P-values shown above each data point. Horizontal bars indicate standard error. Genes categorized as low, medium (med) or high expressed in neurons with medium and high sets partitioned into SATB2+ and SATB- based on genes being present in the SATB2_Hipp gene-set or not.

**Fig 5:**
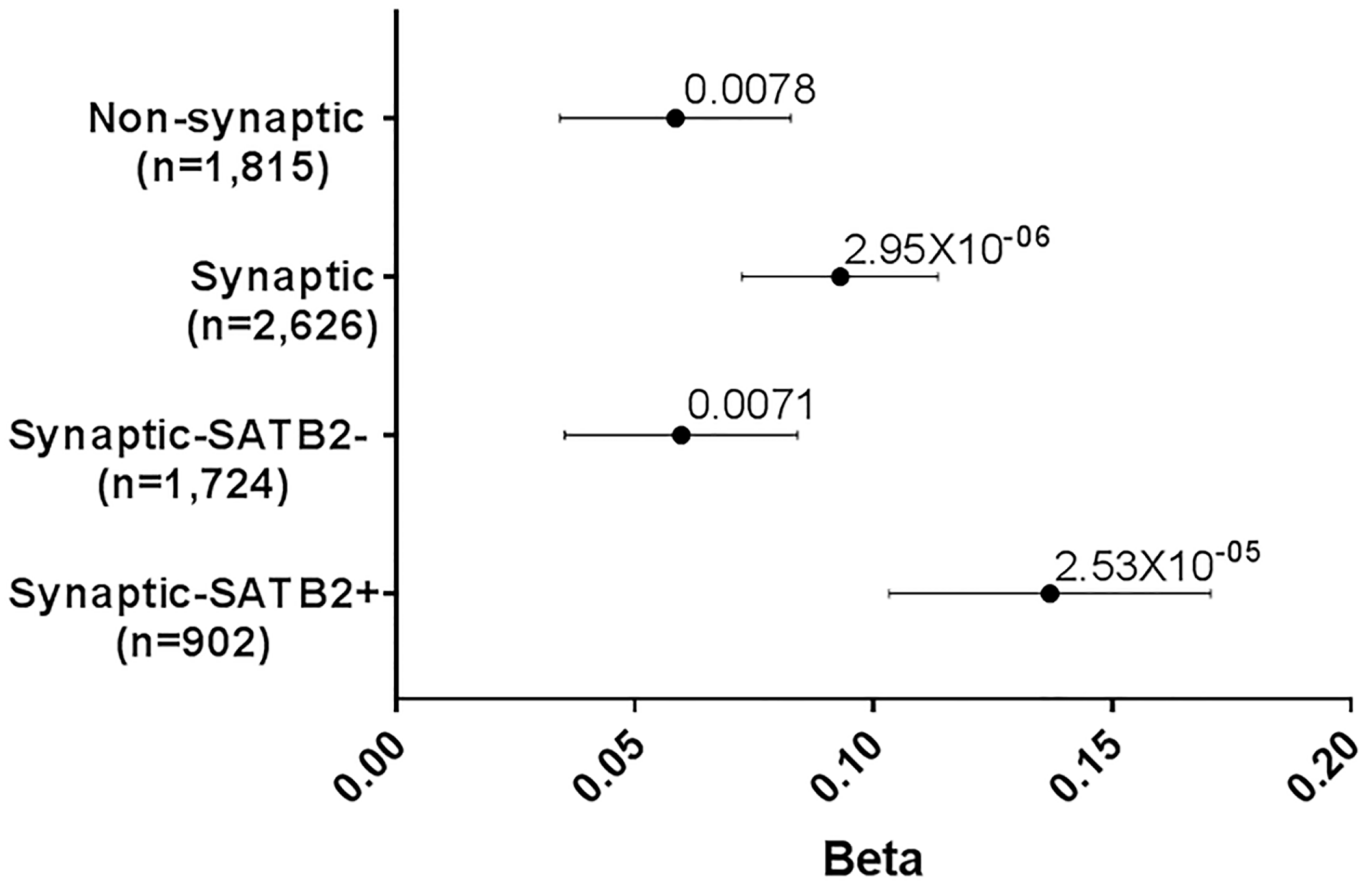
GSA of SATB2_Hipp in EA at the synapse. Gene-sets and number of genes are plotted on the y-axis. Beta values (effect sizes) as calculated by MAGMA are plotted on the x-axis with P-values shown above each data point. Horizontal bars indicate standard error. High expressed genes in neurons were partitioned into those with potentially synaptic functions or not. Those with synaptic functions are further partitioned into SATB2+ or SATB2- based on genes being present in the SATB2_Hipp gene-set or not.

There was no gene expression data available from the primary hippocampal cell cultures to accompany the ChIP-Seq data used to generate the SATB2_Hipp gene-set. To further explore this gene-set, we investigated these SATB2 target genes using data on gene expression levels in (A) the brain, (B) the hippocampus, (C) neurons and (D) at the synapse. (A) Brain expressed genes are strongly enriched for genes associated with EA (P = 1.27x10^-07^) whereas non-brain expressed genes are not (P = 1; Fig 2). When we categorise brain expressed genes into those potentially targeted and regulated by Satb2 or not, i.e. those with or without an adjacent Satb2 binding peak (termed SATB2+ and SATB2-), we observed a much stronger enrichment for EA in the SATB2+ genes (P = 2.11x10^-07^) compared to the SATB2- genes (P = 0.35; Fig 2).

(B) We used data from human hippocampal samples (from the Brainspan Atlas of the Developing Human Brain) at an early stage of post-natal development (37 post conception weeks (pcw) to 1 year) to capture gene expression levels at an equivalent developmental time point to when the primary hippocampal cell cultures were generated and used in the ChIP-Seq analysis. We categorized genes as having a low, medium or high level of expression in the hippocampus. Genes expressed at a medium and high level in the hippocampus show enrichment for EA genes (P = 2.30x10^-4^ and P = 0.0012 respectively; Fig 3). We took these medium and high expressed neuronal genes and categorized them into SATB2+ and SATB2- genes. We observed stronger enrichment for SATB2+ genes compared to SATB2- genes in both the medium and high expressed hippocampal genes (Fig 3).

(C) SATB2 functions in neurons where sets of both medium and high expressed genes show enrichment for EA genes (P = 0.012 and P = 2.67x10^-08^ respectively; Fig 4). We took these medium and high expressed neuronal genes and categorized them into SATB2+ and SATB2- genes. We observed stronger enrichment for SATB2+ genes compared to SATB2- genes in both the medium and high expressed neuronal genes (Fig 4). (D) Given SATB2’s role in synaptic plasticity, we next investigated potentially synaptic genes expressed in neurons. For genes highly expressed in neurons, there was stronger enrichment for EA in those that are potentially synaptic (P = 2.95x10^-06^) compared to those that are not potentially synaptic (P = 0.0078; Fig 5). Categorizing the potentially synaptic genes as SATB2+ or SATB2-, there was stronger enrichment for EA in the SATB2+ genes (P = 2.53x10^-5^) in EA compared to SATB2- genes (P = 0.0071; Fig 5). Together, these data indicate that for genes expressed in the brain, hippocampus, neuron and genes encoding potentially synaptic proteins, those targeted and potentially regulated by Satb2 are contributing more to EA than genes not targeted by Satb2.

We tested SATB2_Hipp for enrichment of genes associated with hippocampal volume [32]. No significant enrichment was observed for SATB2_Hipp in the hippocampal volume GWAS data. Similarly, sets of SATB2+ hippocampal or synaptic expressed genes, which had showed enrichment for EA, did not show enrichment for genes associated with hippocampal volume (S6 Fig).

### Analysis of gene-sets using rare variant data

We studied genes harbouring *de novo* variants identified in patients with SZ, ASD, intellectual disability (ID) and in unaffected siblings and controls [33]. *De novo* variants were categorized as all, loss-of-function (LoF), non-synonymous (NS) and silent and the gene number within each group is detailed in Table 1. Each of the three SATB2 gene-sets contained a significant enrichment of genes containing *de novo* variants for at least one of SZ, ASD or ID, following Bonferroni correction (Table 1). The SATB2+NuRD gene-set was enriched for genes containing *de novo* mutations reported in ASD (both ASD_all, P = 7.00x10^-06^ and ASD_LoF, P = 7.25x10^-04^) and ID (ID_all, P = 5.6x10^-05^ and ID_LoF, P = 8.96x10^-10^). The SATB2_Cort gene-set was enriched for SZ (SCZ_all, P = 3.67x10^-04^), ASD (ASD_all, P = 2.03x10^-04^ and ASD_silent, P = 3.49 x10^-04^), and ID (ID_all, P = 5.08x10^-04^ and ID_LoF, P = 8.63x10^-08^). The SATB2_Hipp gene-set was enriched for SZ (SCZ_all, P = 6.75 x10^-04^) and ID (ID_all, P = 9.86 x10^-04^). Importantly, none of the gene-sets were enriched for genes harbouring *de novo* variants reported in the unaffected or control data. Each of the three gene-sets was significantly enriched for genes listed in the Sys ID database of ID genes (Table 1). Finally, the SATB2_Hipp gene-set was enriched for genes reported to have an excess of disruptive and damaging ultra-rare variants (dURVs) in SZ patients compared to controls (P = 0.0164) based on an exome sequencing study of 12,332 individuals [34].

**Table 1:** Enrichment analysis of gene-sets for genes harbouring d*e novo* variants SZ, ASD and ID, for SysID genes and for genes with an excess of dURVs in SZ.

Gene Group	Gene N	SATB2+NuRD				SATB2_Cort				SATB2_Hipp			
		P-Value	OR	95% CI		P-value	OR	95% CI		P-value	OR	95% CI	
SZ_all	903	0.140	1.66	0.84	3.28	**3.67x10**^-04^*	1.69	1.26	2.25	**6.75 x10**^-04^*	1.31	1.12	1.54
SZ_LoF	111	0.725	1.42	0.20	10.28	0.144	1.76	0.82	3.80	0.109	1.41	0.92	2.17
SZ_NS	576	0.225	1.66	0.73	3.78	0.003	1.70	1.20	2.41	0.003	1.33	1.10	1.62
SZ_silent	216	0.594	1.46	0.36	5.95	0.149	1.54	0.85	2.76	0.202	1.23	0.89	1.69
ASD_all	998	**7.00x10**^-06^*	3.07	1.84	5.14	**2.03 x10**^-04^*	1.69	1.28	2.23	0.060	1.16	0.99	1.36
ASD_LoF	138	**7.25x10**^-04^*	4.83	1.76	13.28	0.375	1.41	0.66	3.03	0.007	1.66	1.14	2.41
ASD_NS	608	0.002	2.74	1.43	5.26	0.039	1.46	1.02	2.11	0.321	1.10	0.91	1.34
ASD_silent	252	0.007	3.24	1.31	8.00	**3.49 x10**^-04^*	2.28	1.43	3.62	0.723	1.06	0.78	1.44
ID_all	158	**5.60x10**^-05^*	5.30	2.14	13.16	**5.08 x10**^-04^*	2.58	1.48	4.49	**9.86 x10**^-04^*	1.77	1.25	2.49
ID_LoF	32	**8.96x10**^-10^*	16.0	4.83	53.47	**8.63x10**^-08^*	7.15	3.08	16.6	0.055	2.02	0.97	4.19
ID_NS	101	0.088	3.20	0.78	13.13	0.083	1.96	0.90	4.24	0.052	1.54	0.99	2.39
ID_silent	25	0.685	6.07	0.82	45.15	0.320	1.01	0.14	7.48	0.058	2.17	0.96	4.90
Unaff/con_all	579	0.442	1.42	0.58	3.49	0.041	1.47	1.01	2.15	0.388	1.09	0.89	1.34
Unaff/con_LoF	56	0.543	2.71	0.37	19.70	0.190	1.95	0.70	5.41	0.633	1.16	0.62	2.17
Unaff/con_NS	368	0.077	2.21	0.90	5.43	0.072	1.52	0.96	2.40	0.272	1.15	0.90	1.48
Unaff/con_silent	155	0.317	0.99	0.14	7.19	0.349	1.41	0.69	2.88	0.865	1.03	0.70	1.53
SysID genes	960	0.001*	2.44	1.40	4.27	**1.08x10**^-06^*	1.99	1.53	2.58	**1.49x10**^-07^*	1.48	1.28	1.72
dURVs in SZ	42	0.597	3.61	0.49	26.43	0.054	2.66	0.95	7.46	0.016*	2.13	1.13	4.01

* Survives Bonferroni correction.

### Functions of risk genes and gene ontology enrichment analysis of gene-sets

S5 Table lists all genome-wide significant genes within the SATB2_NuRD (for SZ or EA), SATB2_Cort (EA only) and SATB2_Hipp (EA only) gene-sets. This is based on MAGMA gene analysis with Bonferroni correction for numbers of genes tested. For the smaller number of genes from SATB2_NuRD and SATB2_Cort, full gene names, their known biology and associated phenotypes are listed in S6 Table. As discussed below, many of these genes have known roles in brain development and are associated with a variety of neurodevelopmental disorders and neurocognitive functions. We performed gene ontology enrichment analysis of EA genes within the larger SATB2_Hipp gene-set (S7 Table). We detected significant enrichment for neuron development and axon guidance.

## Discussion

The SATB2+NuRD gene-set contains genes that were reported to be differentially expressed in the developing neocortex of *Satb2* mutant mice, and genes encoding components of the NuRD complex. The data presented here show that the SATB2+NuRD gene-set is enriched for genes associated with SZ and with EA. It is also enriched for genes harbouring *de novo* variants that have been reported in ASD and ID and for ID genes as listed by SysID. Thus, both analyses of common and rare variants indicate that genes in this set are contributing to the aetiology of SZ, EA and neurodevelopment disorders that involve cognitive dysfunction.

The prenatally-derived SATB2_Cort gene-set is enriched for genes associated with EA. Importantly, the enrichment signal is being driven by those genes that are not only targeted by SATB2 but are also differentially expressed when SATB2 is knocked out. Thus, genes functionally impacted by SATB2 and by extension the processes regulated by SATB2 may represent the molecular mechanisms that underpin EA. Reviewing the genome-wide significant genes for SZ or EA within the SATB2_NuRD and SATB2_Cort gene-sets provides insights into the biological processes that are affected during brain development (S6 Table). A number of the risk genes have known roles in synaptic transmission (*KCNN2* [35], *SLC32A1* [36], *EXOC4* [37]), axon guidance and formation of the corpus callosum (*DCC* [38], *NFIB* [39–41], *BCL11B* [11,16,38,42], *TBR1* [2,43,44]), axon regeneration and neurite branching (*KLF9*[45]), neurite outgrowth and axonogenesis (*FOXP2* [46,47], *NEGR1* [48]), maturation and maintenance of upper-layer cortical neurons (*ATXN1* [49]), cortical cell migration (*AFF3* [50]) and the development of specific sensory circuits in the CNS (*MEF2C* [51], *SEMA6D* [52,53]). Many of these genes are the locations of rare causative mutations for neurodevelopment disorders and have also been associated with neurocognitive functions (S6 Table). This indicates that for these phenotypes, common variants of small effect and rare variants of large effect impact on some of the same genes involved in neocortical organization and circuitry.

Results for SATB2_Cort indicate this gene-set is enriched for genes associated with EA but not SZ. However, analysis of rare variant data shows that this gene-set is enriched for genes carrying *de novo* variants reported for SZ, as well as for ASD and ID. It is possible that there are molecular mechanisms here where common low effect variants are contributing to EA but rare higher effect variants contribute to SZ.

The postnatally-derived SATB2_Hipp gene-set is enriched for genes associated with EA. Further analyses using this gene-set identified a consistent phenomenon: For genes that are expressed in the brain, or hippocampus, or in neurons, or at the synapse, there is an enrichment of genes associated with EA but the effect is stronger for the subsets of genes that are targeted by SATB2. Again, these data indicate that the processes regulated by SATB2 may represent the molecular mechanisms that underpin EA.

There is relatively little overlap of SATB2 targets when comparing SATB2_Cort to SATB2_Hipp. Beyond the temporal and spatial differences in how the ChIP-seq data was produced, and the different experimental procedures used (S8 Table), this also reflects that the role of SATB2 in the postnatal brain differs greatly to its role in the prenatal brain. Compared to the prenatal brain, SATB2 expression extends from the cortex and into the hippocampus and hypothalamus of the adult brain[54]. It plays a crucial role in both long-term and working memory and mediates late long-term potentiation and synaptic plasticity in the postnatal hippocampus [13,14]. SATB2_Hipp is enriched for genes associated with EA, for genes harbouring *de novo* variants for SZ and ID and for genes containing dURVs in SZ, suggesting there is an active pathophysiology in the postnatal brain. At a molecular level, these SATB2 target genes may influence cognitive function via biological processes such as BDNF signalling, epigenetic chromatin modifications and miRNA dysregulation [13]. That impaired fear memory caused by deletion of *Satb2* in pyramidal neurons was successfully rescued through restoration of *Satb2* expression in mouse hippocampus [13] indicates that intervention to restore normal cognitive function may be possible if the molecular mechanisms can be targeted.

EA is a good, though not perfect, proxy for cognitive ability [55] and its specific utility for GWAS is that very large samples have been available for analysis. As new large GWAS for neurocognitive phenotypes, and for SZ, are produced, it will be important to determine if genetic variation within biological processes regulated by SATB2 influence specific traits or instead exert an influence across multiple behavioural and neuropsychiatric phenotypes.

SATB2 is required for the correct formation and structural integrity of regions in the brain such as the cerebral cortex [28], the corpus callosum [11] and the hippocampus [14]. Disrupted expression of SATB2 in these regions can result in anatomical and functional abnormalities associated with a range of behavioural phenotypes [13,14,56,57]. Our analysis of neuroimaging GWAS provided no evidence that SATB2 influences intracranial or hippocampal volume but this too needs further study in larger datasets to determine if SATB2’s influence on SZ risk or cognitive function is mediated via effects on brain structures.

In summary, we have built on single gene associations detected in GWAS of SZ to show that genes that are functionally related to SATB2 and the NuRD complex during neocortical development or are targeted by SATB2 in the pre- and postnatal brain are enriched for common variants associated with SZ and EA, and for rare variants that increase risk of SZ and other neurodevelopmental disorders. These findings are supported by the existing Satb2 mouse models demonstrating deficiency in long-term and working memory upon Satb2 ablation. Thus, this study provides evidence that the molecular mechanisms that underpin SZ and cognitive function include perturbations of the biological processes influenced by SATB2 in the brain.

## Materials and methods

### Ethics statement

Data were directly downloaded from published studies and no additional ethics approval was needed. Each study is referenced and details on ethics approval are available in each manuscript.

### Generation of gene-sets

A study by Alcamo et al. [11] mapped Satb2 expression in developing cortex and showed that Satb2 mutant mice display altered expression of 28 genes associated with axon projection, including BCL11B at E18.5. A more recent study by McKenna et al. [24] performed RNA-seq analysis of cortices at postnatal day P0 to study differentially expressed genes (DEGs) between wild type and Satb2-deficient mice. This led to the identification of 74 DEGs in the deep layers and 15 DEGs in the upper layers of Satb2-deficient cortices. The list of genes from these two studies (n = 117) was increased using data from other studies of Satb2 mouse models [2,15,38]. We also included in this set genes considered to be vital components of the NuRD complex [25] as it has been previously shown to facilitate Satb2-mediated repression of Bcl11b during development [5,12]. Altogether, following conversion of murine gene IDs to orthologous human gene IDs, a total of 127 genes (including SATB2, BCL11B and GATAD2A) were included in this first gene-set named SATB2+NuRD (S1 Table).

The second gene-set was generated using a dataset of 1,341 ChIP-seq peaks (GEO accession: GSE68910) that map binding sites of SATB2 in cortices of wild type mice at E15.5 [24]. ChIP-seq reads were mapped against the mouse NCBI37/mm9 assembly. Functional annotation tool GREAT (http://bejerano.stanford.edu/great/public/html/index.php) was used to associate both proximal and distal input ChIP-seq peaks with their putative target genes and thereby identify genes that may be regulated by SATB2 [58]. We used the default basal plus extension approach within GREAT where each gene in the genome is assigned a basal regulatory domain of a minimum distance of 5kb upstream and 1kb downstream of the transcription start site of the canonical isoform of the gene (regardless of other nearby genes). The gene regulatory domain is extended in both directions to the nearest gene’s basal domain but no more than the maximum extension of 1,000kb in one direction. In addition, GREAT utilizes a set of literature curated regulatory domains that extend the regulatory domain for each gene to include its known regulatory element. GREAT mapped 1,341 ChIP-seq peaks to 1,800 unique gene IDs. For only 144 of these genes, the peak was located 5kb upstream or 1kb downstream of the gene. Given the large default extension region applied in GREAT, this may have led to a number of spurious results. We filtered the peaks mapping to the remaining 1,656 genes by overlapping them with defined enhancers from ENCODE (http://chromosome.sdsc.edu/mouse) to provide extra support for a potential regulatory role. A total of 452 peaks overlapped with mouse brain-specific enhancers (E14.5) and were mapped back to 712 of the 1,656 genes. This resulted in a final set of 856 mouse genes where a SATB2 ChIP-seq peak maps to regulatory regions of those genes. The Ensembl data-mining tool BioMart (http://www.ensembl.org/index.html) was then used to convert these mouse gene IDs to human gene IDs, which resulted in a final set of 778 human genes. This second gene-set was named SATB2_Cort (S2 Table).

The third gene-set was generated using a dataset of 5,027 ChIP-seq peaks (GEO accession: GSE GSE77005) that map binding sites of Satb2 in primary hippocampal cell cultures from wild type mice at postnatal day P0 to P1 [13]. This dataset represents the high-confidence peak list derived from two independent biological ChIP-seq replicates by using the MAnorm to filter out the inconsistent peaks (https://www.ncbi.nlm.nih.gov/pmc/articles/PMC3439967/). We used these ChIP peaks to identify 4,138 human gene targets using the same procedure as mentioned above. This third gene-set was named SATB2_Hipp (S3 Table).

There is a considerable difference in gene number between the SATB2_Cort and SATB2_Hipp gene-sets. Factors contributing to this difference are likely to include the different functions of SATB2 in the pre- and post-natal brain and that the ChIP-Seq data has been generated from different brain regions (cortex v hippocampus). In addition, the SATB2 ChIP-Seq data was generated under different experimental conditions (tissue v primary neuronal cultures) including use of different antibodies (anti-SATB2 v anti V5-tag antibody (ChIP-grade)). These details are supplied in S8 Table.

Sets of ‘brain-expressed’ genes (n = 14,243) and ‘brain-elevated’ genes (n = 1,424) were sourced from the Human Protein Atlas (https://www.proteinatlas.org/humanproteome/brain) and used as covariates in the GSA. Brain-elevated genes are those that show an elevated expression in brain compared to other tissue types.

### GWAS data

Summary statistics from the most recent SZ GWAS [21] were obtained from the Walters group data repository on the MRC Centre for Neuropsychiatric Genetics and Genomics website (http://walters.psycm.cf.ac.uk/). This study included data on 40,675 cases and 64,643 controls. Summary statistics from the most recent EA GWAS [23] were obtained from the Social Science Genetic Association Consortium (SSDAG) website (http://ssgac.org/Data.php, Summary data file: EduYears_Main.txt—discovery and replication cohorts except 23andMe). This study reported results for 328,917 individuals. Summary statistics from a GWAS of hippocampal volume (n = 33,536; [32]) and a second GWAS of intracranial volume (n = 32,438 [29]) were obtained from the ENIGMA Consortium website (http://enigma.ini.usc.edu/). GWAS summary statistics were sourced for AD [59], ADHD (https://www.biorxiv.org/content/early/2017/06/03/145581), ASD (https://www.biorxiv.org/content/early/2017/11/27/224774), BPD [60], CAD [61], CD [62], OCD [63], STR [64], T2D [65] and UC [66].

### Gene-set analysis

A gene-set analysis (GSA) is a statistical method for simultaneously analysing multiple genetic markers in order to determine their joint effect. We performed GSA using MAGMA [26](http://ctg.cncr.nl/software/magma) and summary statistics from various GWAS. An analysis involved three steps. First, in the annotation step we mapped SNPs with available GWAS results on to genes (GRCh37/hg19 start-stop coordinates +/-20kb). Second, in the gene analysis step we computed gene P values for each GWAS dataset. This gene analysis is based on a multiple linear principal components regression model that accounts for linkage disequilibrium (LD) between SNPs. The European panel of the 1000 Genomes data was used as a reference panel for LD. Third, a competitive GSA based on the gene P values, also using a regression structure, was used to test if the genes in a gene-set were more strongly associated with either phenotype than other genes in the genome. The MHC region is strongly associated in the SZ GWAS data. This region contains high LD and the association signal has been attributed to just a small number of independent variants [67]. However, MAGMA still identifies a very large number of associated genes despite factoring in the LD information. Of 278 genes that map to chromosome 6 (25-35Mb), 130 genes were associated with SZ in our MAGMA analysis. To avoid the excessive number of associated genes biasing the MAGMA GSA, we excluded all genes within the MHC region from our GSA of SZ. MAGMA was chosen because it corrects for LD, gene size and gene density (potential confounders) and has significantly more power than other GSA tools [68]. Numerical data used for all figures displaying MAGMA results are provided in S9 Table.

### Analysis of gene-sets using rare variant data

A list of genes harbouring *de novo* variants identified in patients with SZ, autism spectrum disorder (ASD), intellectual disability (ID) and in unaffected siblings and controls were sourced from Fromer et al. [33]. We used the categories of variant as defined in that study (all, loss-of-function (LoF), non-synonymous (NS) and silent; gene number within each group is detailed in Table 1). We sourced a list primary ID genes (n = 960) from the curated SysID database of ID genes (http://sysid.cmbi.umcn.nl/) [69]. From an exome sequencing of 12,332 unrelated Swedish individuals (4,946 individuals with SZ), we sourced a list of 42 genes that had a significant excess of disruptive and damaging ultra-rare variants (dURVs) in SZ cases compared to controls [34]. We performed enrichment analysis of these gene lists with our gene-sets using 2x2 contingency tables with genes restricted to those annotated as protein coding using a background set of 19,424 genes (https://www.ncbi.nlm.nih.gov/). Bonferroni multiple test correction was performed separately for the tests of *de novo* variant genes (n = 48 tests), for the tests of SysID genes (n = 3) and for the tests of dURVs in SZ genes (n = 3).

### Gene expression datasets and potentially synaptic genes

Human brain expression data from the Protein Atlas (http://www.Proteinatlas.org/humanproteome/brain/) was used to filter the SATB2_Hipp gene-set to only include genes expressed in the brain. This dataset included 14,540 genes expressed in, but not unique to, the human brain. For filtering SATB2 gene-sets to include only neuron-expressed genes, we used an RNA-Seq transcriptome and splicing database of glia, neurons, and vascular cells of the cerebral cortex [70]. We used RNA-Seq data from mouse neurons (https://web.stanford.edu/group/barreslab/brainrnaseq.html) and separated genes into three categories; low, medium and high expressed. Low expressed genes were those with Fragments Per Kilobase of transcript per Million mapped reads (FPKM) values <2.0 (n = 12,161 genes). The median FPKM value for the remaining genes was 9.6, hence that was used to categorize medium and high expressed genes; medium (FPKM = 2.0–9.6; n = 5,107 genes) and high (FPKM>9.6; n = 5,189 genes). Mouse gene IDs were converted to human gene IDs using BioMart. For analysis of the SATB2_Hipp gene-set, we used expression data from the hippocampus from pcw 37 to 1 year (n = 4 samples) from the Brainspan Atlas of the Developing Human Brain (http://www.brainspan.org/). We calculated mean expression values and categorised genes as low (FPKM<2.0; n = 9,931), medium (FPKM = 2.0–7.45; n = 5,619) and high expressed (FPKM>7.45; n = 5,842 genes). We followed a method previously outlined [34] to identify potentially synaptic genes.

### Functional annotation

ConsensusPathDB-human (http://cpdb.molgen.mpg.de/) was used to perform overrepresentation analysis of gene-sets and we report on enriched gene ontology-based sets[71].

## Supporting information

S1 Fig Venn diagram showing overlap of genes between SATB2+NuRD, SATB2_Cort and SATB2_Hipp gene-sets. Seven genes (CACNA2D1, MYC, PTPRU, RELN, SKI, TOX and UNC5C) are common to all three gene-sets. Gene symbols for each gene-set and from each overlapping category are listed in S4 Table.
(TIF)

S2 Fig GSA of SATB2+NuRD in SZ, EA plus post-hoc analysis of 10 other GWAS datasets (6 brain-related phenotypes (AD, ADHD, ASD, BPD, OCD and STR) and 4 non-brain diseases (CAD, CD, T2D and UC)). Phenotypes are listed on the y-axis. P-values are shown above each data point, which represent beta values (x-axis). Horizontal bars indicate standard error.
(TIF)

S3 Fig GSA of SATB2_Cort in SZ, EA plus post-hoc analysis of 10 other GWAS datasets (6 brain-related phenotypes (AD, ADHD, ASD, BPD, OCD and STR) and 4 non-brain diseases (CAD, CD, T2D and UC)). Phenotypes are listed on the y-axis. P-values are shown above each data point, which represent beta values (x-axis). Horizontal bars indicate standard error.
(TIF)

S4 Fig GSA of SATB2_Hipp in SZ, EA plus post-hoc analysis of 10 other GWAS datasets (6 brain-related phenotypes (AD, ADHD, ASD, BPD, OCD and STR) and 4 non-brain diseases (CAD, CD, T2D and UC)). Phenotypes are listed on the y-axis. P-values are shown above each data point, which represent beta values (x-axis). Horizontal bars indicate standard error.
(TIF)

S5 Fig GSA of SATB2_Cort in brain volume GWAS data. Gene-sets and number of genes are plotted on the y-axis. P-values are shown above each data point, which represent beta values (x-axis). Horizontal bars indicate standard error. GSA of SATB2_Cort in intracranial volume GWAS data, including the partition of SATB2_Cort genes into those target genes that were DE or not in P0 cortices of SATB2 WT v KO mice.
(TIF)

S6 Fig GSA of SATB2_Hipp in brain volume GWAS data. Gene-sets and number of genes are plotted on the y-axis. P-values are shown above each data point, which represent beta values (x-axis). Horizontal bars indicate standard error. GSA of SATB2_Hipp in hippocampal volume, including hippocampus expressed genes partitioned into SATB2+ and SATB2- and synaptic genes-sets partitioned into SATB2+ and SATB2-.
(TIF)

S1 Table SATB2+NuRD gene-set list. (XLSX)

S2 Table SATB2_Cort gene-set list. (XLSX)

S3 Table SATB2_Hipp gene-set list. (XLSX)

S4 Table Overlap between three SATB2 gene-sets. (XLSX)

S5 Table MAGMA gene-analysis P-values for genes surviving Bonferroni correction in SZ and EA. (XLSX)

S6 Table Full gene names, their known biology and associated phenotypes for associated genes from the SATB2_NuRD and SATB2_Cort gene-sets. (XLSX)

S7 Table ConsensusPathDB over-representation analysis for SATB2_Hipp (Bonferroni significant genes for EA). (XLSX)

S8 Table Information on experimental parameters from previous ChIP-Seq studies that were used to generate the SATB2_Cort and SATB2_Hipp gene-sets. (XLSX)

S9 Table Summary of MAGMA GSA for all gene-sets. (XLSX)
