# Supplementary figures and images for: Genes regulated by SATB2 during neurodevelopment contribute to schizophrenia and educational attainment
Source: PLoS Genet. 2018 Jul 24;14(7):e1007515. doi: 10.1371/journal.pgen.1007515 (PMC6097700; doi:10.1371/journal.pgen.1007515)

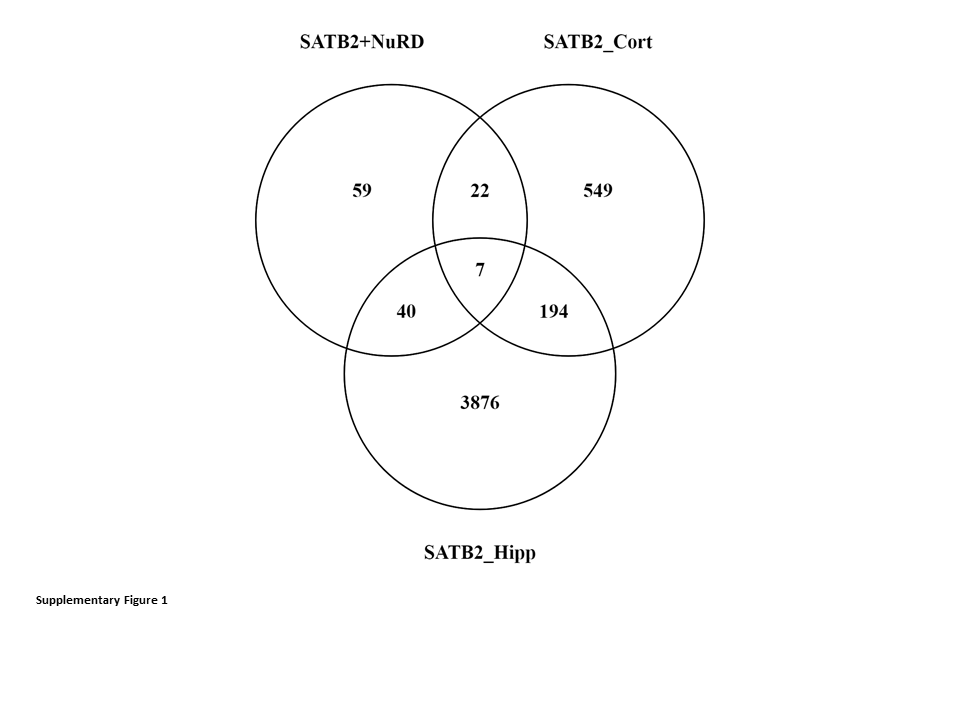

Supplement: S1 Fig — Seven genes (CACNA2D1, MYC, PTPRU, RELN, SKI, TOX and UNC5C) are common to all three gene-sets. Gene symbols for each gene-set and from each overlapping category are listed in S4 Table. (TIF) [file pgen.1007515.s001.tif]

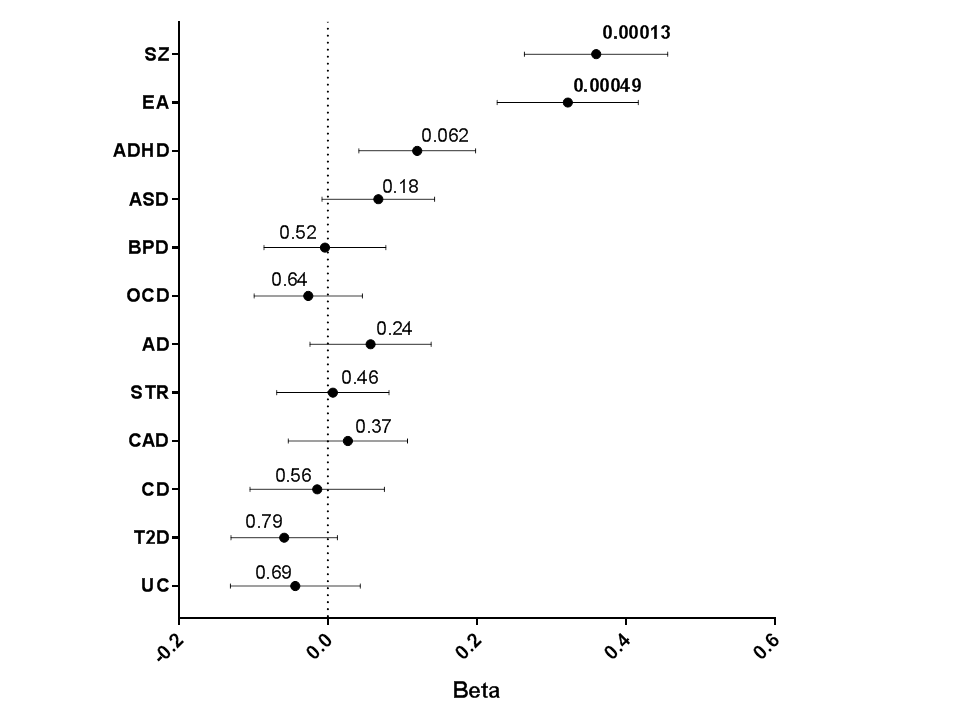

Supplement: S2 Fig — Phenotypes are listed on the y-axis. P-values are shown above each data point, which represent beta values (x-axis). Horizontal bars indicate standard error. (TIF) [file pgen.1007515.s002.tif]

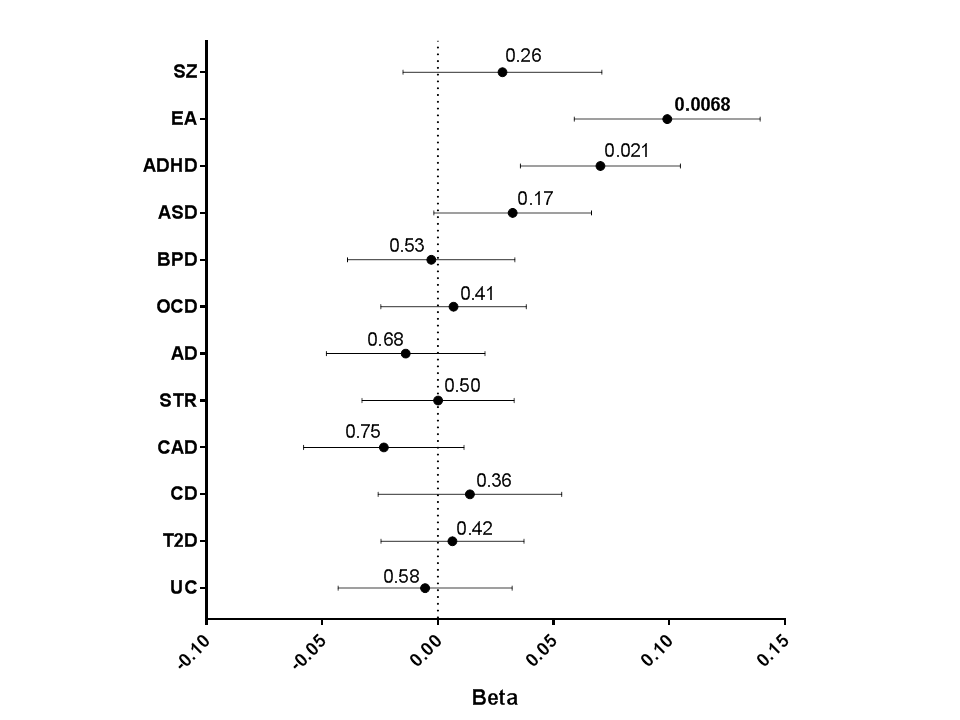

Supplement: S3 Fig — Phenotypes are listed on the y-axis. P-values are shown above each data point, which represent beta values (x-axis). Horizontal bars indicate standard error. (TIF) [file pgen.1007515.s003.tif]

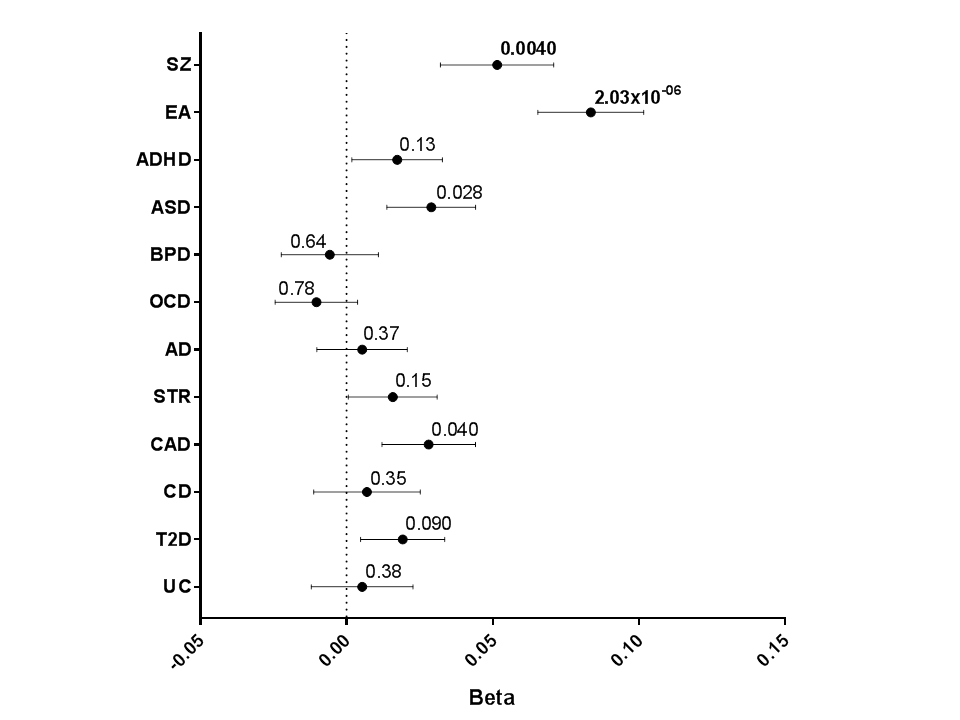

Supplement: S4 Fig — Phenotypes are listed on the y-axis. P-values are shown above each data point, which represent beta values (x-axis). Horizontal bars indicate standard error. (TIF) [file pgen.1007515.s004.tif]

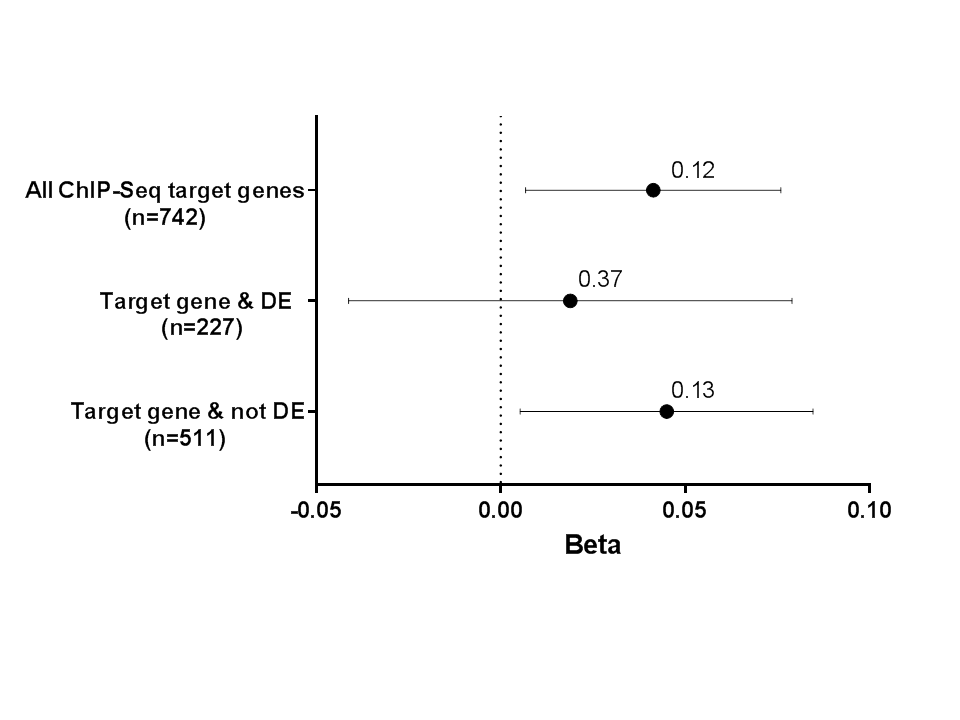

Supplement: S5 Fig — Gene-sets and number of genes are plotted on the y-axis. P-values are shown above each data point, which represent beta values (x-axis). Horizontal bars indicate standard error. GSA of SATB2_Cort in intracranial volume GWAS data, including the partition of SATB2_Cort genes into those target genes that were DE or not in P0 cortices of SATB2 WT v KO mice. (TIF) [file pgen.1007515.s005.tif]

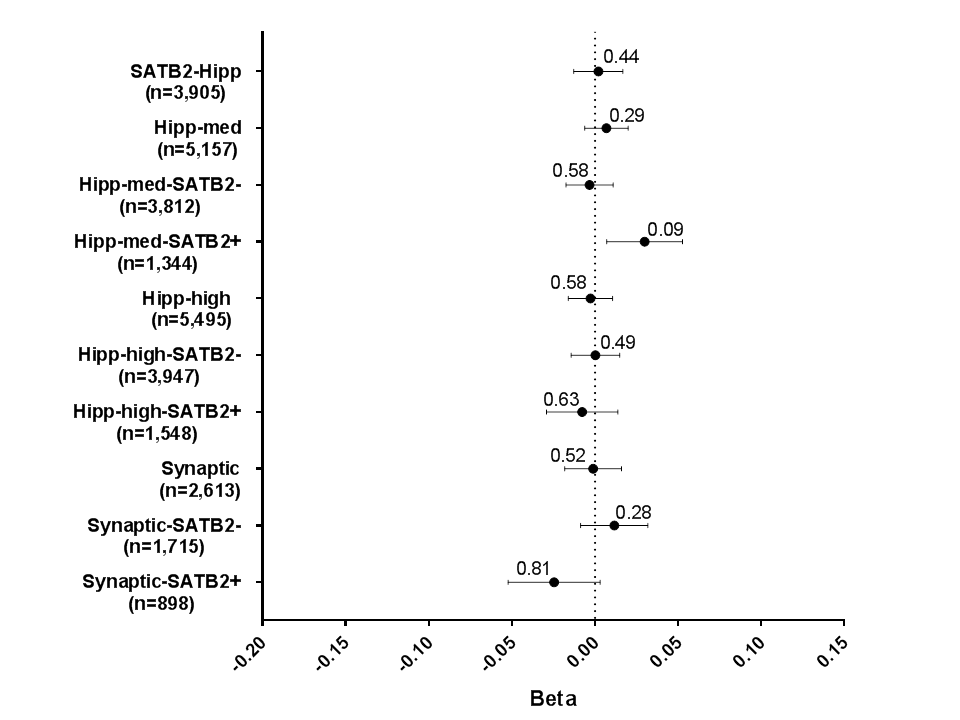

Supplement: S6 Fig — Gene-sets and number of genes are plotted on the y-axis. P-values are shown above each data point, which represent beta values (x-axis). Horizontal bars indicate standard error. GSA of SATB2_Hipp in hippocampal volume, including hippocampus expressed genes partitioned into SATB2+ and SATB2- and synaptic genes-sets partitioned into SATB2+ and SATB2-. (TIF) [file pgen.1007515.s006.tif]
